# A Particle Swarm Optimization-Based Queue Scheduling and Optimization Mechanism for Large-Scale Low-Earth-Orbit Satellite Communication Networks

**DOI:** 10.3390/s25041069

**Published:** 2025-02-11

**Authors:** Ziyong Zhang, Tao Dong, Jie Yin, Yue Xu, Zongyi Luo, Hao Jiang, Jing Wu

**Affiliations:** 1 State Key Laboratory of Space-Ground Integrated Information Technology, Space Star Technology Co., Ltd., Beijing 100095, China; zhangkeke0831@126.com (Z.Z.); dongtaoandy@163.com (T.D.); xubancun509@163.com (Y.X.); zy_luo1@163.com (Z.L.); 2 School of Electronic Information, Wuhan University, Wuhan 430072, China; jh@whu.edu.cn (H.J.); wujing@whu.edu.cn (J.W.)

**Keywords:** large-scale low-Earth-orbit satellite communication networks, time-sensitive traffic, queue scheduling and optimization, particle swarm optimization

## Abstract

The spatial topology of large-scale low-Earth-orbit satellite communication networks is dynamically time-variant, and the load on the output ports of network nodes is continuously changing. The lengths and numbers of output port queues at each network node can affect the packet loss rate and end-to-end latency of traffic flows. In order to provide high-quality satellite communication services, it is necessary to schedule and optimize the lengths and numbers of queues used for transmitting time-sensitive traffic flows at each node’s output port to achieve the best deterministic transmission performance. This paper introduces a queue scheduling optimization mechanism based on the Particle Swarm Optimization algorithm (PSO-QSO) for large-scale low-Earth-orbit satellite communication networks. This method analyzes the relevant parameters of various traffic flows transmitted through the network and calculates the maximum time-sensitive business load within network nodes. It applies the Particle Swarm Optimization algorithm to calculate the optimal solution for the length and number of queues at each node’s output port used for forwarding time-sensitive traffic flows. The mechanism proposed in this paper ensures the deterministic end-to-end transmission of time sensitive traffic in large-scale low-Earth-orbit satellite communication networks and can provide real-time satellite communication services.

## 1. Introduction

Satellite communications have the advantages of wide coverage, high flexibility, and large communication capacity and can avoid the adverse effects of terrain and other factors, supporting real-time, high-quality, and reliable communications in the fields of military, aviation, navigation, and scientific research. Satellite communication will play an important role in the next generation of wireless communication systems [1]. Satellites can be categorized into different types based on their orbital altitudes: low-Earth-orbit (LEO) satellites, medium-Earth-orbit (MEO) satellites, and geostationary orbit (GEO) satellites. Among these, large-scale LEO satellite communication networks, consisting of hundreds or even thousands of LEO satellites with lower orbital altitudes, are designed to provide high-speed broadband Internet connectivity [2]. Large-scale LEO satellite communication networks can offer smaller end-to-end latencies, thus supporting real-time multimedia applications, presenting a broad scope for development [3].

**Figure 1:**
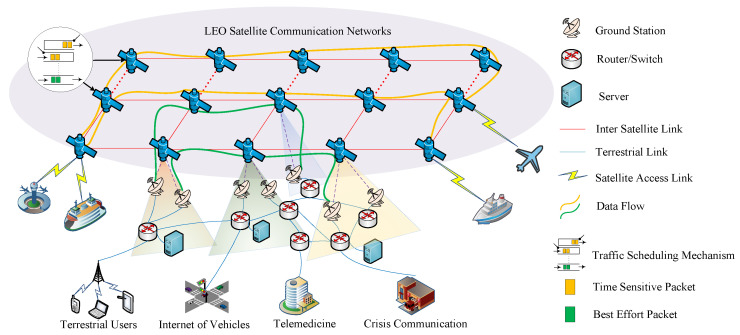
Large-scale LEO satellite communication network architecture.

Compared to terrestrial communication networks, large-scale LEO satellite communication networks have many distinctive features [4]. The spatial scale of satellite communication networks is vast, with limited computing and forwarding capacities [5]. Fortunately, with the advancement of laser communication technology, establishing reliable, high-speed inter-satellite links (ISLs) have become feasible [6]. The propagation delay between LEO satellites is typically in the tens of milliseconds range [7]. Compared to terrestrial networks, large-scale low-Earth-orbit satellite communication networks exhibit higher propagation delays, and their spatial topology presents periodic variability, which results in periodic changes in propagation delay [8]. Furthermore, new traffic flows are continuously injected into the network, resulting in constant changes in the load on the output ports of the nodes within the network. A schematic of a large-scale LEO satellite communication network is shown in Figure 1.

Time-Sensitive Networking (TSN) defines a series of IEEE standards that enhance the capabilities of traditional Ethernet, enabling it to support real-time and low-latency data transmission needs. Currently, its applications have expanded to multiple fields, providing critical network infrastructure support across various industries [9]. Traditional network technologies often struggle to meet the strict requirements for synchronicity, low latency, and high reliability in the aerospace sector.

**Figure 2:**
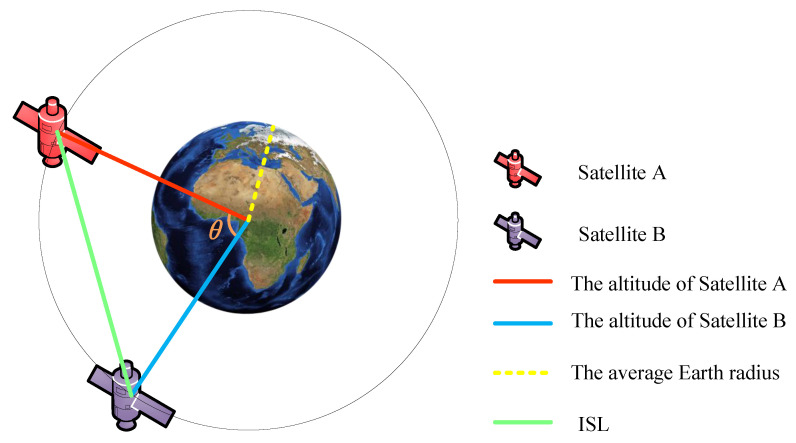
ISL between satellite A and B.

Moreover, the orbital altitude of GEO satellites is 35,786 km, that of MEO satellites ranges from 2000 to 20,000 km, and that of LEO satellites ranges from 500 to 2000 km. The length of an ISL between two satellites can be seen in Figure 2. The average Earth radius is denoted by $R_{E}$. $h_{A}$ and $h_{B}$ are the orbital altitudes of satellites A and B, respectively, and $\left(\lambda_{1},\varphi_{1}\right)$ and $\left(\lambda_{2},\varphi_{2}\right)$ are the sub-satellite point longitudes and latitudes of satellites A and B, respectively. Then, the length of the ISL can be calculated using Equation (1):

(1) $$d_{AB}=\sqrt{{\left(R_{E}+h_{A}\right)}^{2}+{\left(R_{E}+h_{B}\right)}^{2}-2\left(R_{E}+h_{A}\right)\left(R_{E}+h_{B}\right)\cos\left(\theta \right)}$$

where θ is the angle between satellites A and B as seen from the center of the Earth. This angle can be calculated from the longitudes and latitudes of the sub-satellite points:

(2) $$\cos\left(\theta \right)=\sin\left(\phi_{1}\right)\sin\left(\phi_{2}\right)+\cos\left(\phi_{1}\right)\cos\left(\phi_{2}\right)\cos\left(\lambda_{2}-\lambda_{1}\right)$$

From Equations (1) and (2), it can be seen that the length of the ISL increases with the increase in the satellite orbital altitude when θ is fixed. In GEO and MEO satellite communication networks, the high orbital altitudes of the satellites result in longer ISLs, thereby leading to longer propagation delays. Therefore, the demand for low-latency data transmission for time-sensitive services cannot be met. In LEO satellite communication networks, the link propagation delay is relatively short. Applying TSN technology to LEO satellite communication networks can build low-latency, low-jitter communication networks, providing an efficient, reliable, and real-time solution for satellite communication, which helps to improve the overall performance and service quality of satellite communication systems [5].

The Cyclic Queuing and Forwarding (CQF) mechanism is primarily used for traffic control and packet scheduling in network switches or routers [10]. It is considered a key technology for achieving bounded latency transmission in TSN. Its core purpose is to ensure fairness among different traffic flows and efficient forwarding, and it is considered a key technology for achieving bounded latency transmission in TSN. The authors in [11] achieved CQF by injecting timing plans that map time-sensitive flows to underlying resources in both time and space. The DetNet working group extended CQF to Cyclic Scheduled Queuing and Forwarding (CSQF) to support deterministic long-distance transmission. The authors in [12] constructed a large-scale deterministic IP network on actual systems and testbeds of CENI. In [13], the authors addressed the joint routing and scheduling issues of CSQF. In [14], the developers created an agent to support remote cyclic scheduling on profinet and IP protocols. The length of the port queues significantly impacts the network transmission performance based on the CQF mechanism and should be configured flexibly based on the specific network environment, service requirements, Quality of Service (QoS) needs, and the capabilities of network devices. How to reasonably choose the length of the queues is an issue that urgently needs to be addressed [15].

This paper presents a Particle Swarm Optimization-based queue scheduling and optimization mechanism (PSO-QSO) for large-scale low-Earth-orbit (LEO) satellite communication networks with the following innovative contributions:

1. This paper proposes a novel scheduling and optimization mechanism specifically tailored for large-scale LEO satellite communication networks, capable of effectively managing time-sensitive service loads;
2. This paper incorporates the PSO algorithm to optimize the lengths and numbers of the queues within the output ports of each satellite node, obtaining the optimal queue configurations for handling time-sensitive traffic;
3. Through simulation validation, this paper demonstrates that this mechanism can provide the high-quality and efficient transmission of time-sensitive service traffic in large-scale LEO satellite communication networks.

The remainder of this article is organized as follows: Section 2 introduces the queue model of LEO satellites and the impact of CQF queue lengths on transmission performance and describes the principles of the proposed queue optimization mechanism. Section 3 presents the analysis and summary of the simulation results. In Section 4, the advantages of the proposed mechanism and the possible future research directions are discussed. This article is concluded in Section 5.

## 2. Queue Scheduling and Optimization Mechanism of Large-Scale LEO Satellite Communication Networks

### 2.1. CQF Mechanism

The CQF mechanism adopts the gating method from the TAS mechanism, adjusting the state of queues to achieve the forwarding of time-sensitive traffic. CQF utilizes two queues for forwarding time-sensitive service traffic; in a CQF slot, packets in the Open-state queue are forwarded to the next node, while incoming packets are directed to the queue in the Close state. The status of the two queues cycles periodically with the progression of the slot. Within a slot, all packets in the Open-state queue must be completely forwarded.

**Figure 3:**
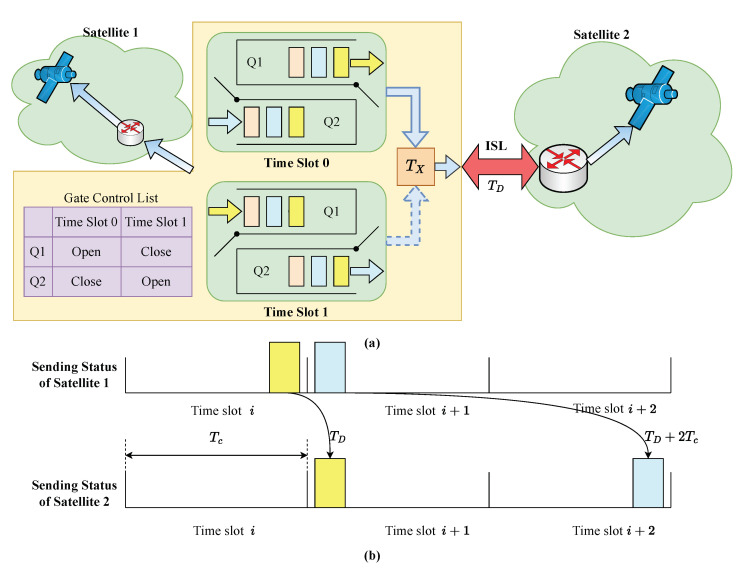
(**a**) Transmission mechanism of CQF in large-scale LEO satellite communication networks. (**b**) Maximum and minimum delay jitter.

If the slot duration is $T_{c}$, the propagation delay is $T_{D}$, and the number of hops for end-to-end packet transmission is *h*; disregarding packet processing delay, the maximum end-to-end transmission delay is then $D_{max}=\left(h+1\right)T_{c}+T_{D}$, and the minimum delay is $D_{min}=\left(h-1\right)T_{c}+T_{D}$. The transmission mechanism of CQF and its maximum and minimum delay jitter are shown in Figure 3.

The CQF mechanism requires that all nodes in the network must complete slot synchronization and strict alignment of slots, and the sum of transmission delay and propagation delay in the network must be less than the length of the slot. To apply the CQF mechanism to large-scale LEO satellite communication networks, this study utilized large-scale deterministic network forwarding technology (LDN). AnLDN assigns a slot ID to each slot at the nodes. In an LDN, when a packet leaves a node’s output port, it carries the sending slot ID of that node. When the downstream node receives this packet, it maps the upstream node’s sending slot ID to its own sending slot ID, and then decides which queue the packet should join based on the sending slot. Therefore, with each node using LDN technology, the processing time is roughly the same, and the need for precision in time synchronization is reduced.

**Figure 4:**
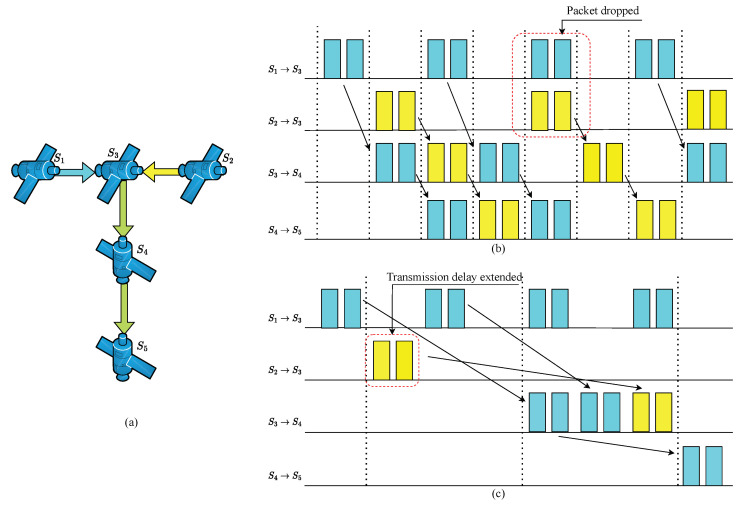
(**a**) A scenario with two time-sensitive flows. (**b**) Transmission packet loss caused by a short queue. (**c**) Transmission time out caused by a long queue.

The length of the CQF queues significantly impacts the forwarding performance of time-sensitive traffic [15]. When CQF queues are too short, although the single-hop queuing delay for traffic transmission is minimal, there is not enough space within the switch ports to buffer more incoming flows, leading to packet loss due to memory overflow. When CQF queues are too long, the single-hop queuing delay for traffic transmission becomes excessively high. This results in an overly long end-to-end transmission delay for the flow, potentially making the flow unschedulable and worsening the network system’s schedulability performance. When there is a high load of time-sensitive traffic on the switch and the maximum tolerable end-to-end transmission delay for various services is short, the traditional CQF mechanism struggles to meet the transmission needs of time-sensitive traffic. Therefore, it is necessary to analyze and optimize the selection of queue lengths and numbers according to the specific scenarios of the network. The influence of the CQF queue length on transmission effect is shown in Figure 4.

### 2.2. On-Board Switch Architecture for LEO Satellites

**Figure 5:**
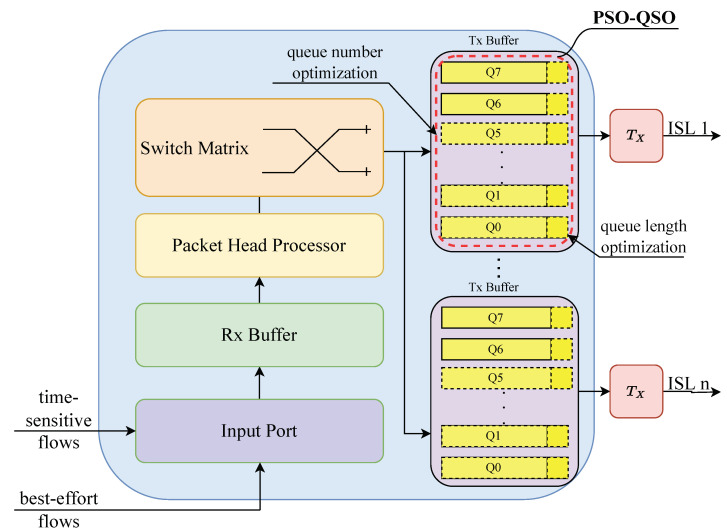
Structure of onboard switches in large-scale LEO satellite communication networks.

In large-scale LEO satellite communication networks, the onboard switches consist of a receiving buffer module, a packet header processing module, a switching matrix, and multiple sending buffer modules for inter-satellite links. Within the switch, each sending buffer module is responsible for the traffic scheduling and forwarding of an inter-satellite link, divided into eight queues (Q0–Q7) according to IEEE 802.1 Qch [16]. We assume that the CQF mechanism is applicable to satellites [17]. For time-sensitive traffic, its packets are stored in Q6 and Q7. For non-time-sensitive traffic, its packets are stored in Q0 to Q5. The structure of onboard switches in large-scale LEO satellite communication networks is illustrated in Figure 5 [17]. As shown in Figure 5, the PSO-QSO mechanism proposed in this paper is applied to optimize the settings of the numbers and lengths of the queues in $T_{X}$ buffers.

### 2.3. Queue Analysis and Optimization Mechanism Based on PSO Algorithm

The traditional traversal algorithm identifies the optimal combination of queue lengths and numbers for the transmission of time-sensitive traffic within each satellite node by trying every possible combination. It requires all possible cases to be calculated sequentially, and the optimal result can only be determined by comparing the results of the calculations for all cases [18]. However, when the total number of satellites in the network is *s*, the computational complexity of the traversal algorithm becomes ${\left(u\cdot n\right)}^{s}$. As the total number of satellites in the network increases, the traversal algorithm has a large amount of computational workload, and the onboard satellite switches will struggle to complete the computation.

The PSO-QSO mechanism proposed in this paper calculates the maximum time-sensitive service load in the network and utilizes the PSO algorithm to optimize the settings of queues at the output ports of each node in the large-scale LEO satellite communication network, obtaining the optimal combination of queue lengths and numbers for transmitting time-sensitive traffic.

In this paper, the maximum transmission unit (MTU) of time-sensitive traffic transmitted within large-scale LEO satellite communication networks is considered to be 1500 bytes. To ensure all packets can be fully accommodated in the queues, the queue length *l* is set as l=MTU·u, where u∈[1,200]. The number of queues *n* is bounded by n∈[2,7]. If optimization calculations are performed frequently, it will lead to high computational costs. The frequent reconfiguration of the onboard switch buffer based on the calculation results will also affect the normal transmission of time-sensitive traffic. Therefore, this paper considers performing a calculation and configuration of queue lengths and numbers every 1000 transmission slots. The loads of time-sensitive traffic arriving at the output ports of each node within the 1000 slots are calculated, and the maximum value is recorded as $L_{peak}$. To obtain the optimal combination of queue lengths and numbers, the settings of queues need to be able to withstand the highest load $L_{peak}$ while keeping the queue lengths and numbers as small as possible. Therefore, the objective function of the PSO can be expressed as

(3) $$f\left(u,n\right)=\left|MTU\cdot u\cdot \left(n-1\right)-L_{peak}\right|$$

In Equation (3), MTU·u·(n−1) represents the product of the lengths and numbers of the queues used to receive time-sensitive traffic, i.e., the total depth of the queues. Detailed steps of the proposed optimization mechanism are as follows.

Firstly, the parameters of the PSO algorithm are initialized. Learning factors $c_{1}$ and $c_{2}$ are used to control the global search and local search ability of PSO. While self learning factor $c_{1}$ is larger, the particle flight trajectory mainly refers to the history information of the particles themselves. While social learning factor $c_{2}$ is larger, the particle flight trajectory mainly refers to the social information of particles movement [19]. In this study, both learning factors $c_{1}$ and $c_{2}$ were set to 1.5. ω is an inertia weight that is initialized typically in the range of [0, 1]. A larger inertia weight facilitates global exploration, and a smaller inertia weight tends to facilitate local exploration to fine-tune the current search area [20]. In this study, ω was set to 0.9 to enhance the search speed for the global optimal solution. In most applications, authors follow the initial suggestion from [21] and restrict the number of particles NP to 20–50 particles. The algorithm proposed in this paper utilizes 20 particles. To improve the search accuracy, this study set the maximum number of iterations to 1000.

The maximum position of each particle is

(4) $$X_{max}=\left(u_{max},n_{max}\right)=\left(200,7\right)$$

The minimum position of each particle is

(5) $$X_{min}=\left(u_{min},n_{min}\right)=\left(1,2\right)$$

The maximum velocity of each particle is $V_{max}=\left(2,2\right)$, and the minimum velocity of each particle is $V_{min}=\left(0,0\right)$.

In this study, the initial positions of each particle in the population were randomly initialized as

(6) $$X_{i}=\left(u_{i},n_{i}\right),1⩽i⩽20$$

The initial velocity of each particle in the swarm is randomly initialized as $V_{i}$. The fitness function value of each particle at the initial position is calculated as

(7) $$p_{i}={\left[MTU\cdot u_{i}\cdot \left(n_{i}-1\right)-L_{peak}\right]}^{2}$$

The historical optimal position of each individual particle is ${\mathrm{pbest}}_{i},1⩽i⩽20$. And the global optimal position of the swarm is gbest.

According to the individual historical optimal position of each particle and the global historical optimal position of the population, the velocity update formula is used to update the velocity of each particle $V_{i}^{\prime }$ and further update the position of each particle $X_{i}^{\prime }$. The velocity update formula can be defined as

(8) $$V_{i}^{\prime }=\omega \cdot V_{i}+c_{1}\cdot rand\left(\right)\cdot \left({\mathrm{pbest}}_{i}-X_{i}\right)+c_{2}\cdot rand\left(\right)\cdot \left(\mathrm{gbest}-X_{i}\right)$$

(9) $$X_{i}^{\prime }=X_{i}+V_{i}^{\prime }$$

where the rand function returns a random number uniformly distributed in the interval (0, 1). The fitness function value of each particle $p_{i}^{\prime }$ is recalculated, and the individual historical optimal position of each particle ${\mathrm{pbest}}_{i}$ and the global optimal position of the swarm gbest are updated. Then, the individual historical optimal position of each particle ${\mathrm{pbest}}_{i}$ and the global optimal position of the swarm gbest are iteratively calculated until the number of iterations reaches $T=10^{3}$. At this time, the global optimal position of the swarm $\mathrm{gbest}=(u_{b},n_{b})$ is the optimal solution for the lengths and numbers of the queues within the output ports of each satellite node in the large-scale LEO constellation for transmitting time-sensitive traffic.

The optimal solution gbest obtained by the optimization algorithm is just enough to withstand the highest time-sensitive traffic load $L_{peak}$. The lengths and numbers of queues can only be positive integers, so $u_{b}$ and $n_{b}$ must be rounded. If the lengths and numbers of queues are rounded down, the total depth of the queues used to transmit time-sensitive traffic is insufficient to withstand $L_{peak}$, resulting in packet loss. To this end, the length and number of queues need to be rounded up. To reduce the packet loss rate of time-sensitive service traffic, $u_{b}$ and $n_{b}$ should be rounded up. This can be defined as

(10) $$U_{b}=\left\lceil u_{b}\right\rceil ,N_{b}=\left\lceil n_{b}\right\rceil$$

Check whether $U_{b}$ and $N_{b}$ meet the following condition:

(11) $$MTU\cdot U_{b}\cdot \left(N_{b}-1\right)-L_{peak}⩾0$$

If the conditions are met, then $L_{b}=MTU\cdot U_{b}$, and $N_{b}$ is the optimal solution for the lengths and numbers of the queues within the output ports of each satellite node in the large-scale LEO constellation for transmitting time-sensitive traffic. The process of optimizing the lengths and numbers of the queues by this mechanism is shown in Algorithm 1.

After optimizing the queue lengths and numbers for scheduling and forwarding time-sensitive traffic flows, the number of queues may exceed two, deviating from traditional CQF forwarding. Similar to CQF, among the n queues designated for scheduling and forwarding time-sensitive traffic, at any given moment, only one queue is in the Open state, referred to as the sending queue; the rest are in the Close state, known as receiving queues. The sending queue is numbered 0, while the remaining receiving queues are sequentially numbered as N=1,2,⋯,n−1. When the time slot switches, the sending queue from the previous time slot, having been emptied, switches its number to n−1 and its state to Close; the numbers of the other receiving queues change to $N^{\prime }=N-1$. Subsequently, the queue with $N^{\prime }=0$ switches its state to Open, becoming the new sending queue for the upcoming time slot. This process repeats in a cyclical manner, resulting in periodic changes in queue status. Therefore, when a packet arrives during the *i*th time slot and enters a queue numbered N, it must wait until the (i+N)th time slot to have a chance for transmission.
**Algorithm 1:** PSO-QSO mechanism.
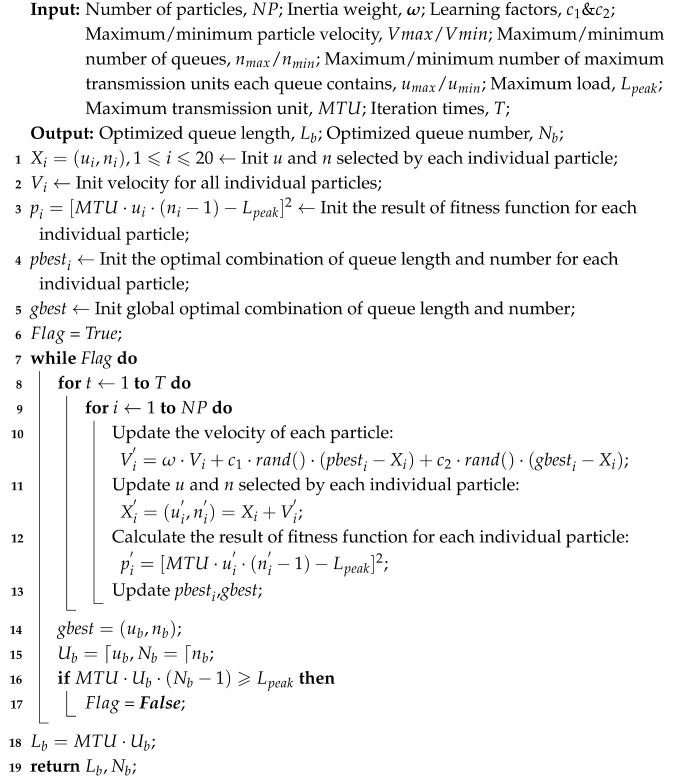


## 3. Results

In this paper, simulation experiments were carried out on the proposed optimization mechanism, and the effectiveness of the proposed mechanism is demonstrated by the analysis of simulation results. The optimization mechanism proposed in this paper optimizes the queue lengths and numbers of the output ports of the onboard switches, mainly acting on the internals of the satellites to reduce the queuing delay. The inter-satellite distances mainly affect the propagation delay and have no impact on the queuing delay. Therefore, the proposed optimization mechanism can be flexibly applied to various configurations of large-scale satellite networks. To simplify the complexity of simulation experiments, the simulation experiments in this study employed the Iridium constellation [22], which is composed of 66 LEO satellites divided into six orbital planes.

**Figure 6:**
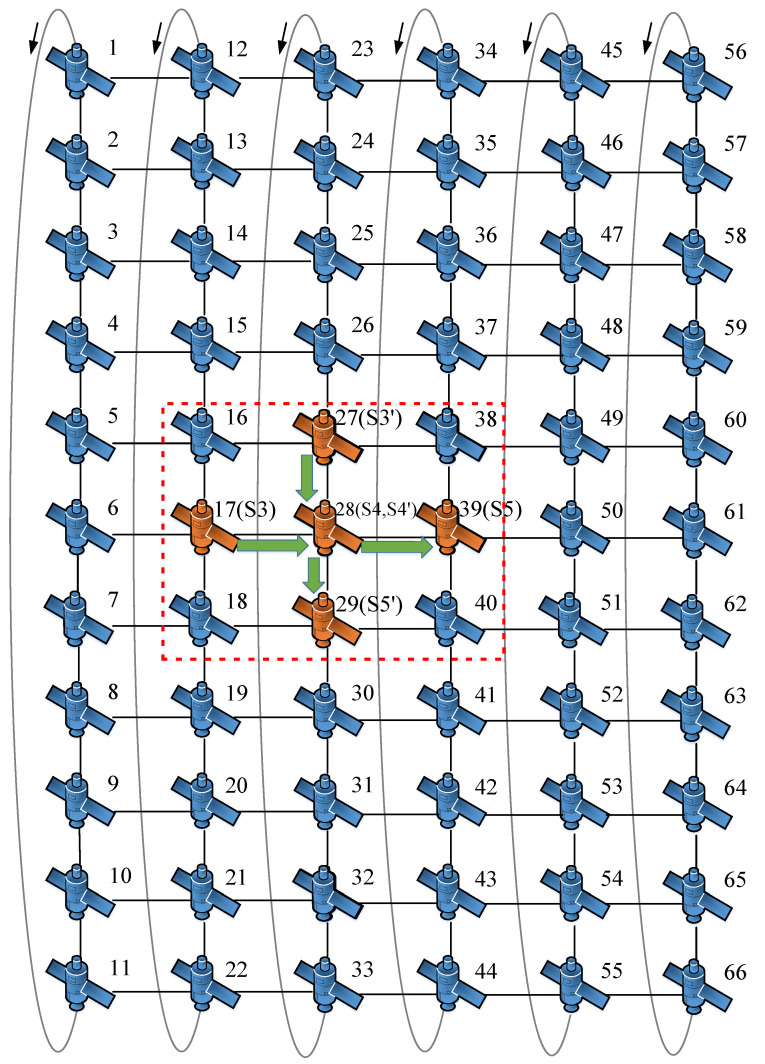
The topology of LEO satellite communication network adopted in this study, where the arrows indicate the directions of the satellite orbits.

The topology of the LEO satellite communication network adopted in this simulation is shown in Figure 6. This paper considers two types of satellite formations:

1. Cluster formation: one satellite in each of three adjacent orbital planes, numbered 17, 28, and 39. They can correspond to S3, S4, and S5 in Figure 4.
2. Trailing formation: three adjacent satellites in the same orbital plane; their numbers are 27, 28, 29. Similarly, they can correspond to S3’, S4’, and S5’ in Figure 4.

For the simulation, we describe a concise scenario to illustrate the behavior of traffic. Satellite communication technology is constantly advancing, especially in the field of inter-satellite links. Thanks to technological innovations such as laser communication systems, higher data rates are becoming increasingly feasible. Therefore, the data transmission rate of the satellite node’s output port was set to R=1 Gb/s, and the transmission slot $T_{c}$ was set to the time it takes for a queue to be completely emptied, that is, $T_{c}=\frac{l}{R}$.

There are various types of services in satellite communication networks, and the packet size may vary greatly. To simulate diverse and realistic traffic, we sample packet sizes from a uniform distribution ranging from 64 to 1500 bytes. This range covers the typical packet sizes of small control signals and large data payloads.

In a dynamic network environment, data traffic is unpredictable. Satellite communication networks often deal with bursty and sporadic traffic patterns, where packets can arrive at any time within a transmission slot. To simulate this randomness, we sampled the arrival time from a uniform distribution ranging from 0 to $T_{c}$.

The main parameters in the simulation are shown in Table 1.

### 3.1. Transmission Delay and Total Queuing Delay of Multi-Node End-to-End Transmission

**Figure 7:**
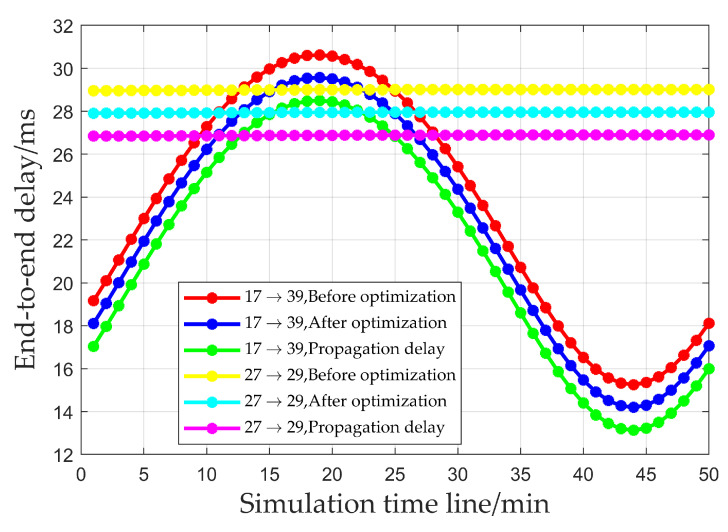
Transmission delay and propagation delay for multi-node end-to-end transmission.

Figure 7 presents the simulation results of the transmission delay and propagation delay for multi-node end-to-end transmission within a single period, under the condition of an unchanged load. From the figure, it can be seen that the optimization algorithm proposed in this paper is capable of reducing the end-to-end transmission delay for multiple nodes, thus enhancing the schedulability of the network. As shown in Figure 7, different satellite formations mainly affect the inter-satellite distances. Therefore, different satellite formations will not affect the performance of this mechanism.

**Figure 8:**
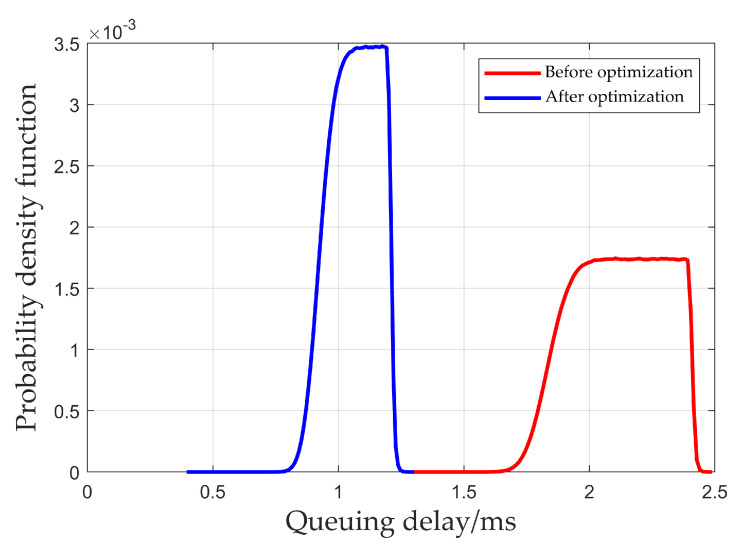
Total queuing delay for multi-node end-to-end transmission.

Figure 8 shows the probability density function of the total queuing delay for multi-node end-to-end transmission within one cycle obtained from the simulation. As shown in the figure, the average queuing delay before optimization is 2.12 ms, and the average queuing delay after optimization is 1.07 ms. The queuing delay after optimization is only 50.47% of the delay before optimization. Thus, the optimization algorithm proposed in this paper can reduce the total queuing delay of packet transmission, thereby reducing end-to-end transmission delay.

### 3.2. Packet Loss Rate of Multi-Node End-to-End Transmission

**Figure 9:**
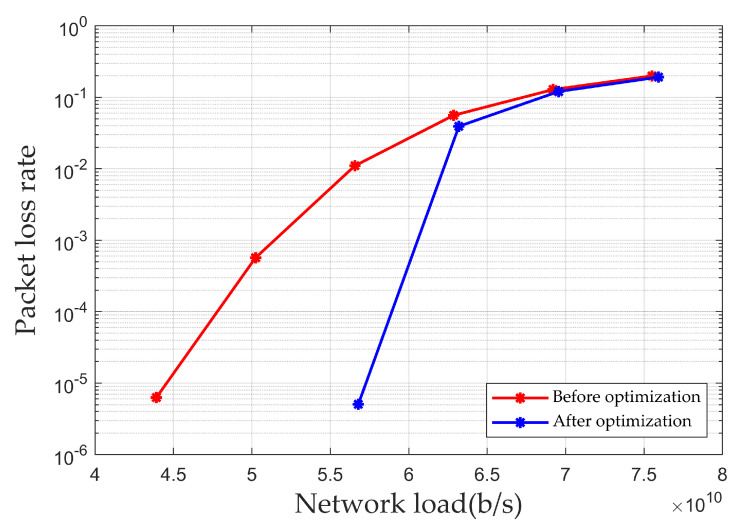
Packet loss rate for multi-node end-to-end transmission.

Figure 9 illustrates the simulation results of the packet loss rate for multi-node end-to-end transmission under different loads. As shown in the figure, when the network load is $5.66\times 10^{9}$ b/s, the packet loss rate before optimization is 1.1%, while the packet loss rate after optimization is only 0.05‰. Thus, the optimization algorithm proposed in this paper can reduce the packet loss rate during end-to-end transmission.

### 3.3. Network Throughput Calculation

**Figure 10:**
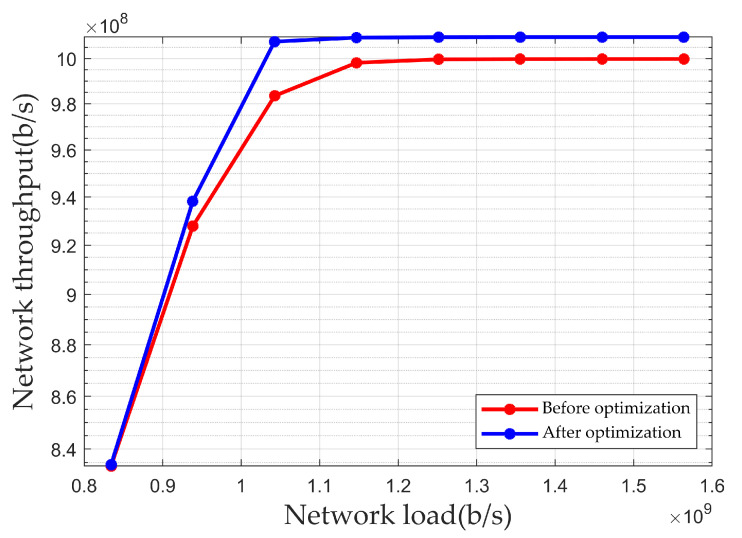
Network throughput under different loads in a scenario without microbursts.

Figure 10 shows the simulation results of the network throughput under different loads in a scenario without microbursts. As shown in the figure, when the network load is $1.04\times 10^{9}$ b/s, the network throughput after optimization has increased by $2.41\times 10^{7}$ b/s compared to the throughput before optimization. Therefore, the optimization algorithm proposed in this paper can increase the network’s throughput, enabling the network to support larger-scale data transmission.

The rate of traffic pouring into a single egress port may exceed the maximum bandwidth of that port, which can cause microbursts. And microbursts are becoming increasingly frequent in high-speed networks. Under the granularity of seconds, network traffic is usually smooth. However, when observed at a finer time granularity, network traffic becomes more bursty. R. Kapoor [23] et al. found through research that on a time scale of 110 to 100 microseconds, traffic exhibits significant burstiness.

**Figure 11:**
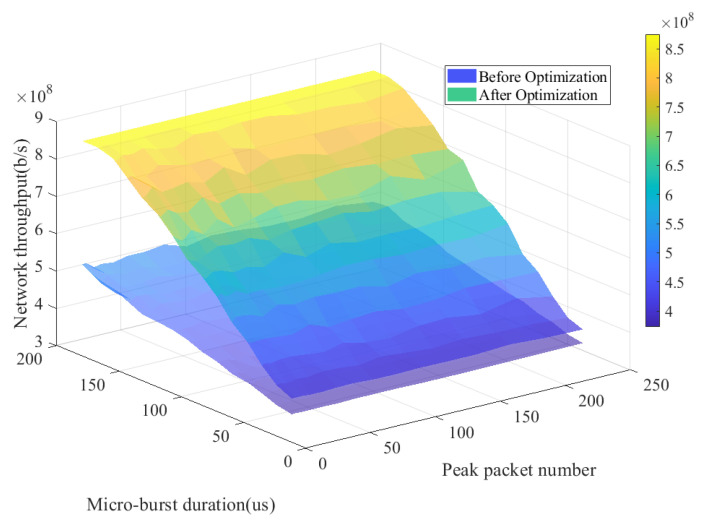
Network throughput for microburst scenarios at different microburst durations and peak loads.

Thus, this study simulated the network throughput in the microburst scenario. Figure 11 presents the simulation results of network throughput in microburst scenarios with different microburst durations and peak loads. It can be observed from the figure that when the microburst duration reaches 200 μs, the optimized network throughput has increased by $3.37\times 10^{8}$ b/s, which is an improvement of 62.72% compared to the throughput before optimization. Therefore, the optimization algorithm proposed in this paper can enhance the network’s throughput during microburst events, mitigating the problem of network congestion in microburst scenarios.

### 3.4. Comparison of Computational Complexity Between Optimization Algorithm and Traditional Algorithm

**Figure 12:**
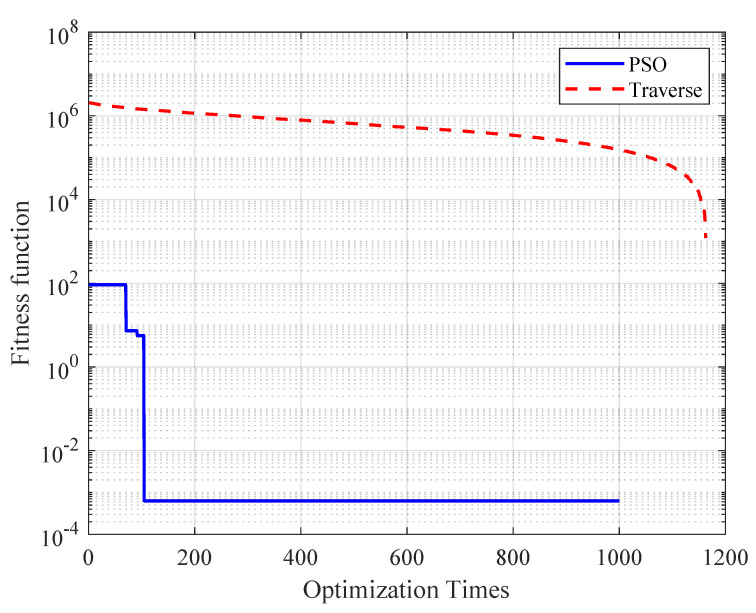
Comparison of computational complexity between PSO algorithm and traversal algorithm.

The optimization algorithm utilizes a particle swarm algorithm to calculate the optimal solution for the length and number of queues in the output ports of the onboard switch, iterating over all possible combinations of queue lengths and numbers. As can be seen in Figure 12, compared to the traditional traversal algorithm, the traversal algorithm needs to calculate all 1200 times to determine the optimal result, while the PSO-QSO mechanism only needs to iterate 105 times to obtain the optimal result. With a time-monitoring program, it takes 6.20 ms for the traversal algorithm to calculate all 1200 combinations, and 0.55 ms for the PSO algorithm to calculate 105 times. The time required for the PSO-QSO mechanism to obtain the optimal solution is only 8.9% of the time required by the traversal algorithm. Therefore, the optimization algorithm has a lower computational complexity and computational overhead.

As shown in Figure 12, there is no improvement in the fitness function for up to 30–40 iterations. These are the initial iterations of the PSO algorithm. In the initial iterations, the particles are exploring the search space widely. The random initialization of particle positions and velocities ensures that the search space is explored from multiple starting points. During this phase, the particles are moving in different directions, and this can lead to periods where the global best position gbest does not change significantly. It is common for the fitness function to show little to no improvement for 30−40 iterations.

The number of particles determines the extent of the coverage of the search space. Increasing the number of particles allows the algorithm to explore the solution space more comprehensively, thereby enhancing the global search capability and increasing the probability of finding the global optimal solution, which is particularly important for complex optimization problems. However, more particles mean more information needs to be exchanged and processed, which reduces the local search efficiency of the algorithm as it approaches the optimal solution, leading to a slower convergence rate. The number of iterations and time required to find the optimal solution will also increase. In considering that the application scenario of the PSO algorithm in this paper is a two-dimensional scenario, the number of particles NP was set to 20 in this study.

## 4. Discussion

From the simulation results above, it is evident that the queue optimization mechanism proposed in this paper can adjust the lengths and numbers of queues used for forwarding time-sensitive traffic, thereby adapting to the actual traffic conditions in the network. Through the application of this mechanism to large-scale LEO satellite communication networks, the packet loss rate and transmission delay for time-sensitive traffic can be significantly reduced. Moreover, this mechanism can also enhance the network’s throughput and better cope with microburst phenomena and has lower computational costs. Future research will further explore traffic scheduling and forwarding mechanisms based on synchronous and asynchronous dynamic adjustment, aiming to reduce delay jitter in the transmission of time-sensitive traffic.

## 5. Conclusions

In this paper, we analyze and study the application of a TSN circular queue forwarding mechanism and propose an optimization mechanism of queue analysis for large-scale LEO satellite communication networks. This mechanism uses the Particle Swarm Optimization algorithm to optimize the maximum time-sensitive traffic load and calculates the optimal solution of the queue length and number of outgoing ports of each node. The simulation results demonstrate that, with the optimization proposed in this paper, the forwarding of time-sensitive flows in the network experiences lower packet loss rates, shorter transmission delays, and higher throughput. This optimization algorithm decreases the effect of microbursts with lower computational complexity. Therefore, this mechanism can improve the effectiveness and reliability of satellite communication and achieve the high-quality and high-efficiency transmission of time-sensitive traffic.

## Figures and Tables

**Table 1:** Parameter table.

Parameters	Value
Total number of satellites (*N*)	66
Satellites in each orbital plane (SN)	11
Number of particles (NP)	20
Maximum number of iterations (*T*)	1000
Learning factors (c1, c2)	1.5
Inertia weight (ω)	0.9
Maximum position of each particle ($X_{max}$)	(200, 7)
Minimum position of each particle ($X_{min}$)	(1, 2)
Maximum Transmission Unit (MTU)	1500 bytes

## Data Availability

The data that support the findings of this study are available from the corresponding author upon reasonable request.
